# Mild-to-wild plastic transition is governed by athermal screw dislocation slip in bcc Nb

**DOI:** 10.1038/s41467-022-28477-4

**Published:** 2022-02-23

**Authors:** Q. Rizzardi, C. McElfresh, G. Sparks, D. D. Stauffer, J. Marian, R. Maaß

**Affiliations:** 1 Department of Materials Science and Engineering, University of Illinois at Urbana-Champaign, Urbana, IL 61801 USA; 2 Department of Materials Science and Engineering, University of California, Los Angeles, Los Angeles, CA 90095 USA; 3 Bruker Nano Surfaces & Metrology, Hysitron Products, Eden Prairie, MN 55344 USA; 4 Federal Institute of Materials Research and Testing (BAM), Unter den Eichen 87, 12205 Berlin, Germany

**Keywords:** Mechanical properties, Metals and alloys

## Abstract

Plastic deformation in crystals is mediated by the motion of line defects known as dislocations. For decades, dislocation activity has been treated as a homogeneous, smooth continuous process. However, it is now recognized that plasticity can be determined by long-range correlated and intermittent collective dislocation processes, known as avalanches. Here we demonstrate in body-centered cubic Nb how the long-range and scale-free dynamics at room temperature are progressively quenched out with decreasing temperature, eventually revealing intermittency with a characteristic length scale that approaches the Burgers vector itself. Plasticity is shown to be bimodal across the studied temperature regime, with conventional thermally-activated smooth plastic flow (‘mild’) coexisting with sporadic bursts (‘wild’) controlled by athermal screw dislocation activity, thereby violating the classical notion of temperature-dependent screw dislocation motion at low temperatures. An abrupt increase of the athermal avalanche component is identified at the critical temperature of the material. Our results indicate that plasticity at any scale can be understood in terms of the coexistence of these mild and wild modes of deformation, which could help design better alloys by suppressing one of the two modes in desired temperature windows.

## Abstract (web summary)

Recent studies have recognized that plasticity in metals can be controlled by dislocation avalanches. Here the authors examine temperature-dependent microplasticity in bcc Nb and reveal the dominance of athermal screw dislocation activity during intermittent slip.

## Introduction

Metals with a body-centered cubic (bcc) crystal structure are the basis of an important group of structural metals in engineering applications, such as ferritic steels and many refractory alloys. The plastic flow behavior of bcc metals is strongly influenced by temperature, which, unlike metals with face-centered cubic (fcc) or hexagonal close-packed (hcp) structures, has important implications for their mechanical behavior^1,2^. This temperature dependence is generally ascribed to the thermally activated nature of screw dislocation motion, which is based on kink-pair nucleation and glide^3–6^. The standard model of plasticity for bcc metals implicitly assumes that the behavior of the dislocation network can collectively be determined from the properties of a single screw dislocation. This picture assumes a laminar plastic response consisting of uncorrelated dislocation dynamics and well-defined plastic averages. However, recent evidence pointing to correlated dislocation behavior is causing a paradigm shift in our understanding of metal plasticity towards the importance of spatiotemporally heterogeneous plastic flow^7–10^. This type of correlated dislocation activity, referred to as dislocation avalanches, is found to be scale-free and consequently cannot be characterized by average quantities. Long-range elastic coupling at a scale much beyond the minimum structural length-scale, the lattice parameter in a metal, controls these collective defect physics and leads to statistical signatures of slip sizes that follow fractal behavior with sizes up to the micro-meter scale^11–13^ and that may propagate across grain boundaries^14^.

Here we exploit the temperature sensitivity of rate-limiting screw-dislocation mobility in bcc metals to show how a reduction in thermal energy increasingly suppresses the length scale of dislocation avalanches. With this gradual change, the statistical description of the dislocation avalanches transitions from a scale-free-like truncated power-law to a scale-dependent exponential form. Our results further show that the stress-strain response can be separated into strain increments that are either thermally activated or essentially dynamically athermal. This bimodal behavior is found to emerge due to a fundamentally different temperature sensitivity of smooth plastic activity that corresponds to uncorrelated dislocation motion in comparison to intermittent dislocation avalanches responsible for the athermal contribution. Remarkably, we can unveil that the athermal plasticity component is dominated by otherwise temperature-sensitive screw-dislocation glide. This finding adjusts our classical approach of describing bcc plasticity on the basis of individual thermally activated screw segment motion, and we find that the non-thermal collective screw activity is caused by the emergence of local stresses much in excess of the Peierls stress, ${\tau }_{P}$. The fraction of athermal slip activity decreases markedly with decreasing temperature, indicating a reducing screening length, thereby ‘quenching out’ the long-range interactions required for collective dynamics. Temperature-dependent small-scale testing in combination with state-of-the-art three-dimensional (3D) discrete dislocation dynamics (DDD) simulations of Nb microcrystals are used to reveal these fundamental discoveries.

## Results

### Microplastic stress–strain behavior of Niobium between 173 and 368 K

We begin with studying the stress–strain response of Niobium (Nb) specimens in the temperature range between 173 and 368 K. This range includes the critical temperature, ${T}_{{\rm {c}}}$ (ca. 290–320 K^15,16^), below which plastic flow is nominally controlled by the mobility of screw dislocations. Here *T*_c_ is defined as the temperature at which screw and edge dislocations have comparable mobilities due to sufficient thermal activation of the screw dislocations^15–17^. This coincides with the point at which the bulk flow stress becomes insensitive to a further increase of the testing temperature and the mobility of both screw and edge dislocations begins to equalize. A direct consequence of the mobility mismatch between screw and edge dislocations below *T*_c_ is the strong increase in yield or flow stress with decreasing temperature—a characteristic generally not present in metals with fcc or hcp crystal structure.

**Fig. 1:**
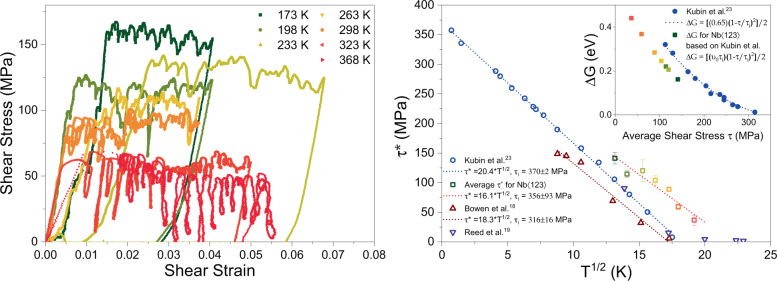
Temperature-dependent microplastic flow of Nb. **a** Compressive shear-stress shear-strain curves obtained at a displacement rate of 0.6 nm s^−1^ on Nb $\langle 123\rangle$-microcrystal at temperatures ranging between 173 and 368 K. **b** Shear stress at which events occur along the curves in **a** as a function of ${T}^{1/2}$ (represented by the squares). The error bars correspond to the standard deviation. Literature data from refs. ^18,19,23^ is also included for comparison, all of which follow the same trend based on the used model by Kubin et al.^23^. The Inset shows the calculated barrier energy based on the same model.

Figure 1a displays shear-stress vs. shear-strain curves for nominally identical Nb single crystal specimens with a diameter of 2 μm and an aspect ratio of 1:3. The loading axis was oriented along the single-slip $\left\langle 123\right\rangle$-orientation. All curves exhibit a size-dependent stress increase in comparison to a bulk reference crystal (~100 MPa at 298 K^18,19^), as well as stress–strain instabilities (abrupt increments of strain), due to stochastic collective dislocation rearrangements. In between such intermittent stress–strain discontinuities, smooth quasi-static flow behavior is observed, indicating that the total strain can be decomposed into a fraction attributed to smooth plasticity of quiescent dislocation glide and a fraction generated by dislocation avalanches^20^. In the following, we will demonstrate experimentally that both fractions respond strikingly differently to changes in temperature.

The flow stress increases with decreasing temperature and can be well captured with classical models that distinguish between an internal (${\tau }_{{\rm {i}}}$) and an effective thermal stress (${\tau }^{* }$), the sum of which expresses the total shear stress, $\tau$, required to deform the crystal^21,22^. It is the increase of the thermally-activated component ${\tau }^{* }$ with decreasing temperature that captures the increasing resistance to motion of screw dislocations. Using ${\tau }_{{\rm {i}}}-\tau =-{\tau }^{* }=A\times {T}^{1/2}$^23^, an essentially Arrhenius-type transition-state theory allows determining the relevant thermal-activation parameters, namely, the effective barrier energy, $\Delta G$, and the activation volume, defined as ${v}^{* }=\partial \Delta G/\partial \tau$. In the absence of appreciable hardening^10^, we can calculate an average constant flow stress along the full deformation curve for each temperature. Figure 1b shows results for ${\tau }^{* }$ as a function of temperature (expressed as $({T}^{1/2})$) and for $\triangle G$ as a function of $\tau$ (inset to Fig. 1b) obtained in this work as well as earlier literature data for bulk single crystals^18,19^. Despite the larger scatter and the expected stress-scale offset, both of which are characteristic for small-scale crystal deformation, the microcrystal data displays the anticipated stress-temperature scaling. $\Delta G$ is determined to be ca. 0.3 eV at 298 K and is therefore in excellent agreement with bulk plasticity of Nb when using the average flow stress^15,24^.

### Intermittent microplasticity of Niobium between 173 and 368 K

**Fig. 2:**
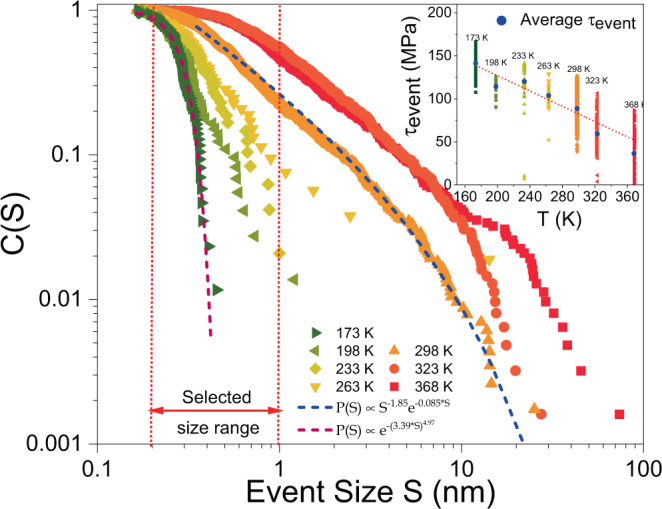
Temperature-dependent avalanche statistics. CCDF (size distribution) of events for temperatures ranging between 173 and 368 K. The indicated event size range where events have been recorded for all temperatures is subsequently used for the analysis show in Fig. 3. The inset shows the average shear stress for the selected size range for all temperatures.

A much different scenario emerges when we now consider that the stress–strain response is composed of abrupt displacement jumps and smooth plastic flow. These two contributions allow us to conduct a strain-increment partition, meaning we can separately evaluate smoothly accumulated strain and collective dislocation activity that is channeled via avalanches and that punctuate the different displacement increments. We extract the avalanche size $S$ using Wiener filtering followed by thresholding, as described in refs. ^25,26^ and outlined in more detail in the “Methods” section. More specifically, $S$ represents the axially resolved net displacement generated during an avalanche. How the event size $S$ relates to the shear displacement or shear strain is described in the “Methods” section. Once an event is identified, the stress at which it occurs is also determined. Figure 2 shows the stress-integrated complementary cumulative distribution function (CCDF), $C(S)$, for different temperatures. A power-law scaling of type $C\left(S\right)\propto {S}^{-\alpha }{e}^{-\lambda S}$ is found to capture the data at $298$, $323,$ and 368 K using the maximum-likelihood estimation approach^27,28^. With decreasing temperature, the distributions shift to smaller values, indicating the gradual loss of large shear offsets via avalanching. This effect of temperature on the event-size statistics is, e.g., in stark contrast to hcp crystals, for which the critical dynamics underlying the power-law scaling was not observed to change in a temperature range from $253$ to 270 K ^29^. At the lowest temperature investigated here (173 K), the power-law scaling is lost, and an exponential distribution (specifically a Weibull distribution) becomes the appropriate statistical model. As the temperature decreases, there is thus a change from correlated, scale-free dislocation activity to a collective dislocation activity with a well-defined scale (~0.3 nm, the mean of the exponential distribution at 173 K). The scaling of the mean avalanche stress-versus-temperature (inset to Fig. 2) is that of the average flow stress. We have thus here revealed how a process that at higher temperatures is controlled by scale-independent correlated dislocation dynamics upon cooling becomes scale-dependent, where the emerging length-scale at the lowest probed temperature is of the order of the Burgers vector modulus, i.e., the inter-atomic distance.

**Fig. 3:**
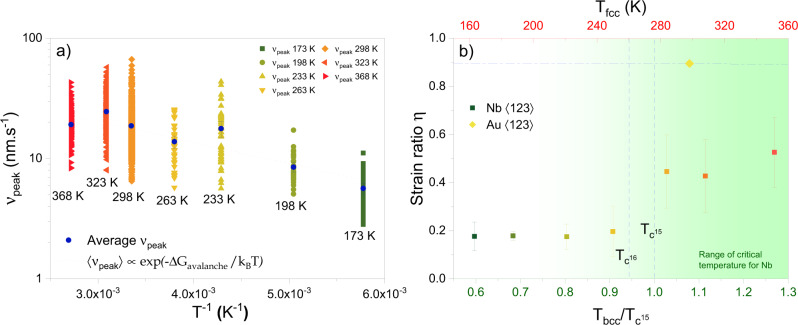
Athermal and thermally activated components in microplastic flow of Nb. **a** Peak velocity of events for temperatures ranging between 173 and 368 K. An exponential fit is applied to determine the effective barrier energy. The barrier energy is found to be an order of magnitude lower than the expected value for the flow stress in bcc crystals. **b** Temperature-dependent strain ratio with indicated literature values of *T*_c_ for Nb^15,16^. The contribution of strain from avalanches increases around *T*_c_, eventually reaching a value more in line with single-slip oriented gold. The error bars correspond to the standard deviation for each temperature.

In conjunction with this temperature-dependent change in intermittency, we also find that the dislocation avalanches are, much in contrast to the average flow response of the crystal and the classically expected thermal-activation scheme, almost insensitive to changes in temperature. Figure 3a highlights this via an Arrhenius construction that plots a measure of rate (represented by the peak velocity ${v}_{{{\rm {peak}}}}$ of the collective dislocation event^30,31^) as a function of inverse temperature. A detailed account for how ${v}_{{{\rm {peak}}}}$ is determined can be found in earlier work^26,30–32^ and some key aspects of the conducted data processing are outlined in more detail in the Supplementary Online Materials (SOM). Individual data points spreading out vertically are the corresponding data points within the size range indicated in Fig. 2. This range was chosen because ${v}_{{{\rm {peak}}}} \sim {S}^{n}$, where $n$ is some scaling exponent that has been derived theoretically^32–34^ and that also has been revealed experimentally^12,26,30,31,35^. This power-law relation means that larger events are faster. To ensure a meaningful assessment of ${v}_{{{\rm {peak}}}}(T)$, all data sets need to therefore span the same range in $S$, which is the highlighted size-range in Fig. 2. Figure 3a also shows the average of each data set and an Arrhenius fit that returns an effective mean-barrier energy of 0.04 eV. This is clearly an order of magnitude smaller than that obtained from the averaging of the smooth stress–strain segments and also the classical barrier energies of bulk crystals. Thus, the motion of dislocation avalanches in the Nb microcrystals is practically athermal, which shows that plasticity proceeds bimodally: (1) via thermally activated dislocation activity that within the resolution of the experiments generates a continuous increase in plastic strain, and (2) via athermal and collective dislocation rearrangements that contribute with abrupt increments in strain. We emphasize that this athermal character emerges when evaluating the spatiotemporal avalanche dynamics, whereas the net event size $S$ is sensitive to temperature. This indicates that decreasing temperature does not quench the motion of the abrupt collective rearrangement, but instead amplifies pinning sites within the lattice that control the avalanche arrest.

The proportion of this split between thermally activated and athermal dislocation activity can be expressed as a plastic strain rate ratio $\eta =\frac{{\varepsilon }_{{\rm {a}}}}{{\varepsilon }_{{\rm {t}}}}$, where ${\varepsilon }_{{\rm {a}}}$ is the summed plastic strain of all avalanches and ${\varepsilon }_{{\rm {t}}}$ the total plastic strain. $\eta$ is observed to change with decreasing temperature, as shown in Fig. 3b. Thus, the contribution of dislocation avalanches to the total plastic strain decreases markedly with decreasing temperature, indicating that the dislocation ensemble loses its correlated collective behavior. With a similar starting value for the dislocation density (all samples originate from the same bulk crystal), we understand this to be a consequence of an increasing dominance of the Peierls (free) energy, which we outline in more detail in the “Discussion” section.

As seen in Fig. 3b, for the experiments conducted here, the relative dominance of ${\varepsilon }_{{\rm {a}}}$ increases at around 95–100% of the ${T}_{{{\rm {bcc}}}}/{T}_{{\rm {c}}}$ ratio, where ${T}_{{{\rm {bcc}}}}$ is the bcc-crystal testing temperature and ${T}_{{\rm {c}}}$ the critical temperature. Now the rate-limiting dislocation process occurs at a comparable time scale to the experiment, therefore allowing $\eta$ to rise markedly. Since this pronounced change in $\eta (T)$ for the Nb microcrystals coincides very well with the temperature range reported for ${T}_{{\rm {c}}}$, our results seem to offer a direct experimental approach to determining ${T}_{{\rm {c}}}.$ Whilst not covered at higher temperatures, we expect that $\eta (T)$ will gradually continue to increase until eventually a similar level as for a typical fcc material (shown for a $\left\langle 123\right\rangle$-oriented Au in Fig. 3b) is reached.

### Gaining insight into the key slip mechanisms using DDD simulations

The present experimental results provide crucial access to fine scale features of the deformation microstructure, but at the same time several puzzling observations call for a deeper investigation. In particular, the weak temperature dependence of the avalanche-controlled plastic behavior is at odds with a classical picture of screw-dominated, thermally activated, plastic response in bcc metals. As indicated earlier, edge dislocations can measurably participate in slip at temperatures above ${T}_{{\rm {c}}}$, and thus one could conceivably postulate a partition between edge—which display a weak inverse temperature behavior^36^—and screw dislocation contributions. Indeed, recent studies in bcc systems where edge dislocations are assumed to exist and to be stabilized in confined volumes in nanocrystals suggest an enhanced edge dislocation role in plastic deformation^37^. To this end, we perform three-dimensional DDD simulations using the MODELib package^38^ in specimens of equal dimensions and crystal orientation as the experimental samples. See the “Methods” section for further details on the simulations.

While the temperature range explored in the simulations coincides with the experimental conditions, the high spatial and temporal resolution of DDD simulations makes simulating quasistatic strain rates impractical. In fact, our simulations were performed under strain-control at uniaxial compressive strain rates of 7180 and 718 s^−1^. At first, such a disparity in deformation rate may appear too large to allow a meaningful comparison with the experimental data. However, a number of recent studies suggest that materials displaying similar strain-rate sensitivity (SRS)—even when studied by different methods—may be governed by identical deformation mechanisms^39,40^, which thus makes them suitable for direct intercomparison. Therefore, as a necessary verification of the reliability of comparisons between experimental observations and simulation results, we investigate the SRS exponent, $m={\left(\frac{\partial {{{{{\rm{ln}}}}}}\sigma }{\partial {{{{{\rm{ln}}}}}}\dot{\varepsilon }}\right)}_{\varepsilon ,T}$, and its dependence on temperature separately with both approaches. Supplementary Fig. 1 displays $m\,{{{{{\rm{vs}}}}}}.\,T$ for both the DDD simulations and the experimental tests, together with data from the literature for bulk specimens. Clear linear dependences can be observed, with proportionality constants of $2.9\times {10}^{-4}$ (DDD) and $3.1\times {10}^{-4}$ (in experiments), in very good agreement with one another. In addition, these values also agree with prior results for both bulk single crystals and polycrystals that typically are in the 2–3 × 10^−4^ interval^24,41,42^. Such good agreement, then, enables a cautious but direct comparison between the experimental results and the DDD simulations to reveal the fundamental mechanisms underpinning the deformation tests.

**Fig. 4:**
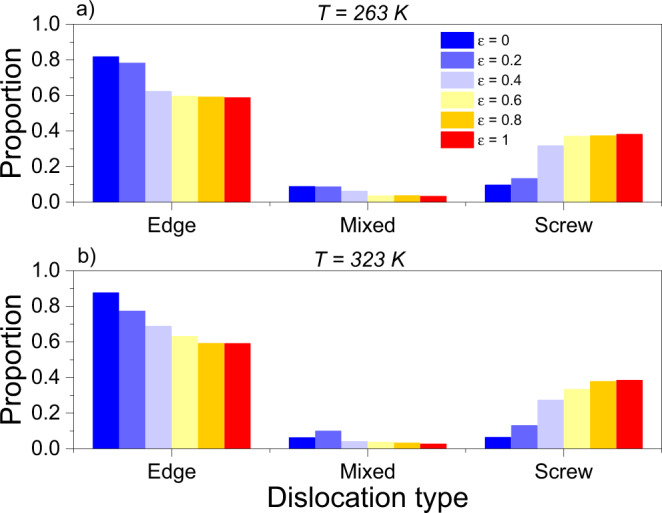
Strain- and temperature-dependent fractions of dislocation types. As strain is increased during simulations at both **a** $263{{{{{\rm{K}}}}}}$ and **b** $323{{{{{\rm{K}}}}}}$, a number of edge dislocations rotate towards a screw configuration and the system eventually stabilizes to a proportion of roughly 40% screw, 60% edge dislocations, and a near-insignificant proportion of mixed behavior.

At both investigated strain rates, the simulations display only minor changes in $C(S)$ (Supplementary Fig. 2a), suggesting temperature-independent avalanche statistics over the considered temperature range, but a distinct flattening of $C(S)$ is seen with increasing rate (Supplementary Fig. 2b). This is a known feature in the rate-dependent avalanche regime and is a result of a coupling between the applied rate and the dynamic behavior of dislocations^43^. In the following, we focus on data obtained at 718 s^−1^ and begin with interrogating the DDD simulations to understand the reasons behind temperature-insensitive avalanche statistics. As mentioned above, a predominance of edge dislocations in avalanche dynamics would naturally lead to temperature insensitivity, but as shown in Fig. 4 for both $263$ and $323{{{{{\rm{K}}}}}}$, even when the initial dislocation network is highly skewed towards edge character, rotation of these into screw orientations prior to yielding leads always, irrespective of the initial dislocation structure, towards an increase in the overall screw character of the network at the expense of edge components.

**Fig. 5:**
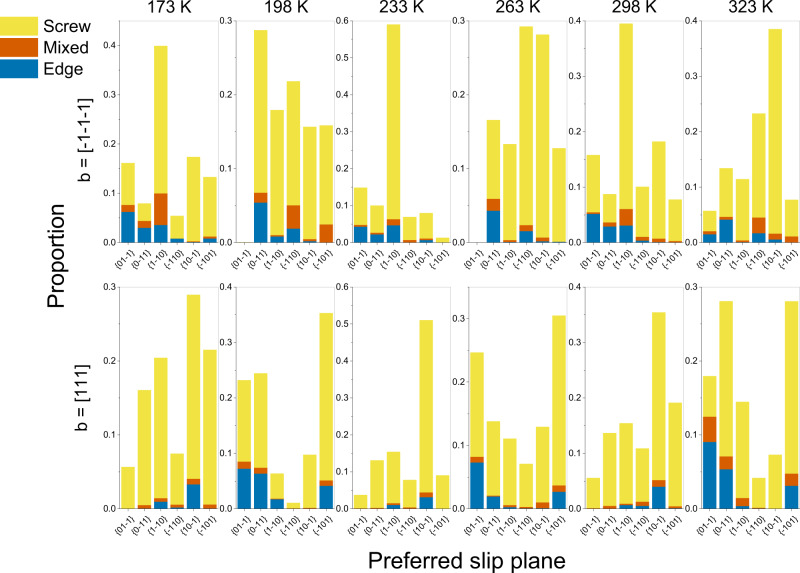
Temperature-dependent fractions of dislocation types across slip systems. There is no clear trend as to which slip planes are preferentially activated for dislocation avalanches, as can be expected in bcc crystals. However, at all temperature ranges and regardless of the preferred slip plane, the vast majority of dislocations identified to participate in avalanche behavior are screw dislocations.

A subsequent identification of the slip systems with the highest plastic distortion rate identifies the $[111]$ and the $[1\bar{1}\bar{1}]$ slip directions as decidedly the most dominant ones. Filtering out the subset of dislocation segments involved in avalanches with these slip directions reveals that screw dislocations are unquestionably the most prominent segment types, irrespective of temperature. Figure 5 conclusively reveals a clear predominance of screw segments for dislocations with ½$\left[111\right]$ and ½$[1\bar{1}\bar{1}]$ Burgers vectors across all slip planes and temperatures. This is a remarkable finding, as it connects the observed weak temperature dependence typical of nonscrew segments to a dislocation microstructure unequivocally dominated by screw dislocations, which, as such, would be expected to be highly susceptible to temperature.

However, this observation is also at odds with the observed tendency towards equipartition between screw and nonscrew segments calculated in Fig. 4 for the whole system. Thus, an unexplained puzzle linking athermal behavior with screw-dislocation slip predominance exists at temperatures and strain rates at which screw dislocation motion would indisputably be expected to be thermally activated.

**Fig. 6:**
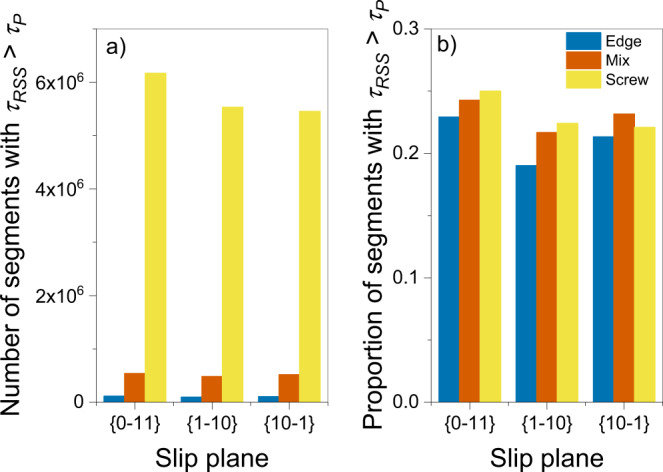
Distribution of segments with stresses exceeding the Peierls stress. **a** Number of segments subjected to stresses that exceed ${\tau }_{\rm {{P}}}$. The dominance of screw dislocations in the stress landscape above ${\tau }_{{\rm {P}}}$ agrees with the proportions seen in Fig. 5. **b** Proportion of segments above ${\tau }_{{\rm {P}}}$ for each dislocation behavior type. This shows that the fundamental difference in dislocation behavior stems more from the availability of segments of a particular orientation than a fundamental difference in their activation.

The answer to this puzzle lies in the heterogeneous nature of the dislocation microstructure (casting further doubts on the use of averaging to characterize dislocation networks in deformed specimens). Indeed, we have analyzed the spatially resolved shear stress distribution in all the DDD samples and have found a distinct correspondence between shear stresses well in excess of the Peierls stress and the location of screw dislocation pileups in the computational specimen. Figure 6a gives the number of screw dislocation segments subjected to stresses above ${\tau }_{{\rm {P}}}$ compared to other non-screw segments at 173 K, and Fig. 6b displays the proportions of segment types that are experiencing stresses above ${\tau }_{{\rm {P}}}$. As the results indicate, screw segments are overwhelmingly subjected to stresses $\tau \, > \, \tau _{{\rm {P}}}$. This suggests that avalanches are essentially controlled by bundles of screw dislocations with internal local stresses above the Peierls stress. However, the key implication of this observation is that above ${\tau }_{{\rm {P}}}$ screw dislocations display a viscous motion much in the manner of their edge counterparts^44,45^, i.e., non-thermally activated. In other words, by overstressing screw dislocation pileups, their thermal sensitivity becomes neutralized and that is what is manifested at the specimen level in the stress–strain curves.

## Discussion

Much of the current thinking about plasticity in bcc metals is predicated on two fundamental axioms: (i) that a clear separation of screw and edge segments exists due to wide differences in their mobility, and (ii) that—at least at low-to-intermediate temperatures— the material response can be explained in terms of the properties of isolated screw dislocations. Indeed, earlier work on dislocation avalanches in bcc metals has revealed an extended dislocation-avalanche velocity-relaxation^26^, suggesting screw-dominated avalanches at room temperature. Given this screw-dominance during avalanching, one would consequently expect that correlated long-range collective motion would be highly susceptible to temperature below *T*_c_. However, we conclusively show here that, in fact, the contrary is found when working in confined volumes. Building onto previous acoustic-emission measurements that revealed the dominance of intermittent (wild) plasticity in hcp crystals but not in fcc, where continuous (mild) and wild plasticity events coexist^46^, the present experiments follow this terminology but now allow a separate investigation of the mild and wild contributions to plastic flow as a function of temperature in bcc lattices. In doing so, it becomes apparent that the dislocation dynamics underlying intermittent plasticity are temperature insensitive despite being dominated by screw dislocations. We find that high local stresses enable this unexpected non-thermal screw dynamics. Above ${T}_{c}$ and with increasing temperature, the dominance of screw dislocations during correlated dislocation activity will naturally vanish. This transition does not occur gradually; rather, our experiments reveal an abrupt intensification of the intermittent plasticity component at *T*_c_, suggesting a direct and straightforward experimental way for its determination.

**Fig. 7:**
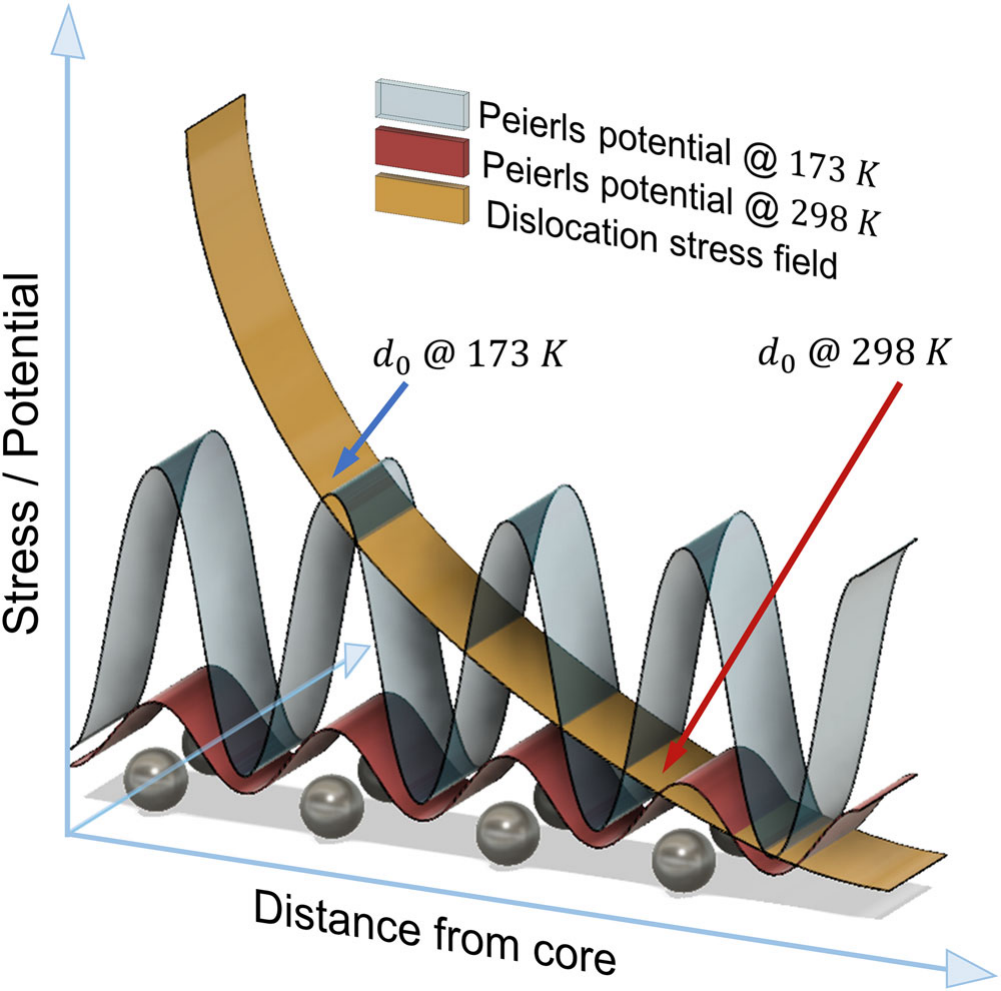
Impact of Peierls potential on dislocation-dislocation coupling. For a given dislocation density, the value of ${d}_{0}$ (which is a function of the thermal energy of the system) dictates the maximum interaction distance of a given dislocation in its local stress landscape and dislocation density. At 173 K, coupling of dislocations can only occur over a much smaller range than at 298 K because of different Peierls potentials, which increasingly favors individual dislocation motion with decreasing temperature.

In contrast to the screw-dominated but athermal avalanche dynamics, a continuous suppression of the corresponding net strain increment (event size $S$) is revealed with decreasing temperature (Fig. 2). This indicates that while the dynamics remains athermal, the increasing lattice friction does arrest avalanches quicker—a trend that can be rationalized when considering an interplay between the elastic coupling distance of a dislocation and the lattice friction. For this, we consider a distance ${d}_{0}$ that expresses the scale beyond the dislocation core at which elastic coupling is suppressed due to the Peierls potential^47^. The dominance of the latter increases with decreasing temperature, thereby decreasing ${d}_{0}$ upon further cooling. Thus, at a comparable average dislocation spacing, the mutual interaction distance via simple elastic coupling of dislocations is limited to $2{d}_{0}$, beyond which the lattice friction dominates. This means that low temperatures result in small ‘screening’ distances $2{d}_{0}$ that favor individual dislocation behavior. Reaching a sufficiently high temperature will eventually lead to much larger $2{d}_{0}$ than the average dislocation spacing of the system, therefore allowing correlated activity. Figure 7 provides a qualitative diagram describing the effect of the Peierls potential on the reach of a given dislocation’s stress field and the gradual suppression of its capacity for long-range interaction as the temperature decreases, all assuming a constant dislocation density. We note that Fig. 7 solely is a schematic representation for a given dislocation density, aiming at displaying the interplaying effects of the energy landscape of the lattice (Peierls potential) and the stress field due to an isolate dislocation at different temperatures.

Then, assuming a strain-independent dislocation density for microcrystal deformation^48^, one can thus conclude that the effective screening distance $2{d}_{0}$ reduces with cooling, being a result of an increasing lattice friction. Viewed differently, this temperature-dependent suppression of the event size *S* is analogous to introducing a sufficiently strong field of pinning points that drive the transition from critical scale-free like to non-critical exponential-like avalanche size statistics^49^. Here, this pinning is of course exerted by the lattice itself, rather than being a result of a solute or second phase distribution, as is typical in engineering alloys.

This transition from critical scale-free like to non-critical exponential-like avalanche size statistics, as seen in Fig. 2, represents a clear reduction in the net length scale associated with dislocation activity and raises the question of whether a further decrease of the testing temperature would push the statistics to even smaller scales. Experimentally, this regime is not accessible due to the sensor limitations discussed in the SOM, but since the measured length scale is a far-field signature of line-defect motion, one cannot exclude the possibility of subtle rearrangements at even lower temperatures that in sum contribute an $S$-value smaller than the experimental limit in this work. Specifically, one can consider the activity of single-armed spiral sources in the deforming microcrystals. The motion and interaction of such dislocation segments have been shown to be the dominant mechanisms in finite-sized crystals^50–52^, where the revolution of one segment would contribute with one Burgers vector shear displacement. It is thus possible that numerous segments are activated at very low temperatures, but that their individual motion via kink-pair nucleation is limited such that the net shear displacement amounts to less than the Burgers vector magnitude. A verification of this scenario would, however, require a much higher displacement resolution than current state-of-the art nanomechanical testing can provide.

Even though avalanches are screw-dominated below *T*_c_, this does not exclude the involvement of other segment types. In fact, edge dislocations and mixed segments are expected to couple to the rearrangements induced by screw-dominated avalanches, although their contribution to the total admitted strain by avalanches appears to be negligible. Our good agreement between simulation and experiments below *T*_c_ indicates that deformation is always conducted at a too high rate relative to the relaxation time scale of the screw-network. In the limit of infinitely slow deformation, or at least much below the average relaxation time of the screw activity, the dominance of athermal screw activity observed here during avalanching must thus be lost. However, considering the slow deformation rate of 0.6 nm s^−1^, this is beyond realistic means, underlining the general validity of our observation beyond microplasticity.

At the bulk scale, these aspects of bcc plasticity would never have been unraveled because of the ample availability of other rate-limiting processes that can occur at lower local stresses. Restricting the space of configurations that dislocations can achieve has thus allowed us to isolate highly stressed screw dislocation pileups that upon depinning decouple from the temperature-controlled Peierls potential and that exhibit an effective barrier energy that is about one order of magnitude smaller than the classical thermal-activation picture provides. This separation in athermal wild and temperature-sensitive mild parts of glide demonstrates the importance of considering dislocation ensembles, rather than basing the description of plasticity on the behavior of single dislocations.

## Methods

### Sample preparation

All experiments were conducted on a single crystal of commercially pure niobium with a total usable surface area of ~9 mm^2^. Electron backscatter diffraction (EBSD) characterization confirmed the crystal to be oriented $\langle 123\rangle$. Microcrystals of cylindrical shape, height of 6 μm and diameter of 2 μm, were carved in the crystal via focused ion beam (FIB) milling in a FEI Helios scanning electron microscope (SEM) dual beam SEM-FIB system. The final tapering angle along the length of each microcrystal was evaluated at <$1^\circ$. Each microcrystal was carved with a crater-like zone around it 35 μm in diameter to allow mechanical loading of the microcrystal without contacting the bulk of the crystal.

### Microcompression setup and testing

The microcrystals were mechanically stressed under uniaxial compression with the use of a flat punch head affixed to a triboindenter of brand Bruker-Hysitron, the response dynamics of which was outlined in ref. ^31^. Two different compression setups were used. Experiments at temperatures ranging from 173 to 273 K were conducted in a TI-980 indenter with an 18 μm diameter flat punch at the Bruker-Hysitron laboratory in Eden Prairie, MN. The TI-980 was equipped with a cooling stage and temperature control unit, thus allowing the stabilization of low temperatures during the experiments. Experiments at temperatures ranging from 298 to 368 K were conducted in a TI-950 indenter with an 11 μm flat punch at the Materials Research Laboratory at the University of Illinois at Urbana-Champaign, IL. As above, the TI-950 was equipped with a heating stage and temperature control unit to stabilize the experiment temperature and limit thermal drift. Compression experiments were displacement rate-controlled, which means that the microcrystal was set to advance against the transducer at a constant rate of 0.6 nm  s^−1^, for a total displacement per experiment of 150 nm (or was stopped after recording an event contributing a total displacement at or above 150 nm), which corresponds to a strain rate of ${10}^{-4}\,{{{\mbox{s}}}}^{-1}$. Drift levels were consistently recorded as ≤0.3 nm s^−1^. The processing unit of both indenting setups is a Performech II with a manufacturer specified minimum displacement resolution of 0.006 nm, which means this is the minimum depth difference that can be recorded between two consecutive datapoints. The noise threshold for event detection was determined to be 0.165 nm, a value comparable to what was reported in ref. ^7^. The SOM outlines the noise determination and experimental resolution in more detail. The total compression displacement for each microcrystal was set as 1500 nm, i.e. 10 experiments per microcrystal, for a final strain of $\sim 25 \%$. The data acquisition rate was limited by the indenter’s internal memory but was at least 800 Hz for all experiments. All analyzed abrupt strain increments due to dislocation avalanches represent a net axial displacement change of the microcrystals. This is referred to as an event size $S$ in the main text. Given the known crystal orientation and the active slip planes, it is possible to convert $S$ into a corresponding total shear offset (or shear magnitude) via a geometric factor. SEM investigation of the microcrystals after compression confirm that the slip planes preferred by the crystals are the ones predicted by Schmid’s law, namely $\{211\}\langle \bar{1}11\rangle$. This leads to the geometric factor $S={d}_{\tau }\times {\mathrm sin }\theta$, where ${d}_{\tau }$ is the shear offset and $\theta$ the angle between the normal to the activated slip plane and the loading axis, itself normal to the top surface of the sample where compression is applied. In this case we have $\theta \approx 40.2^\circ$ and $S={0.645d}_{\tau }.$ In other words, the quantity event size $S$ and the shear offset express the net length change of the sample in different ways. Whilst these quantities would numerically be different than $S$, all trends remain the same.

### Characterization

Beyond the initial EBSD work used to confirm grain orientation, high-resolution SEM pictures of each microcrystal were taken before and after each experiment in order to visualize the slip line formation following compression.

### Analysis

The data output of the experiments are simple arrays containing time, depth, load, and corresponding voltages. All data files were processed via Matlab with the following process: first, the moving slope of depth over time is established to detect potential jumps in the depth trace that correspond to intermittent dislocation behavior. Each instance is reviewed manually to determine if the jumps correspond to actual shifts in displacement or simply noise (the noise detection routine is described in more detail in the SOM). Once identified, these mechanical traces are bound in time to determine a start and an end between which a velocity profile is calculated. The trace is then smoothed by Wiener filtering, to remove any potential subpeaks due to remaining noise in the datapoints outside the bounds of a given event. This allows the determination of a peak velocity of the filtered trace, and from there the time bounds of the event are redefined at the points corresponding to the full width at 10% of the peak maximum. The resulting events have a filtered depth, timespan, and velocity profile. Finally, they are combined into large datasets, over which we can apply statistical analysis tools, such as the CCDF. All distribution fitting was performed in Python using the powerlaw package developed by Jeff Alstott^53^, which uses a maximum-likelihood estimation (MLE) method. All figures except for Fig. 7 were created in OriginLab (while Fig. 7 was created in AutoDesk Fusion 360).

### DD simulation

**Fig. 8:**
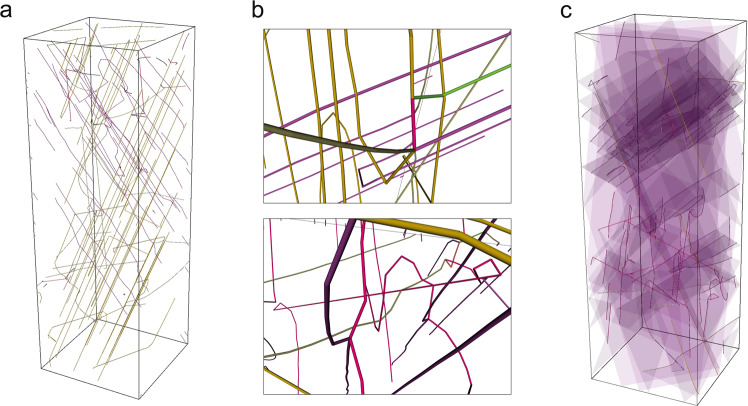
Examples of discrete dislocation dynamics simulation box. **a** Entire microcrystal after deforming to $\varepsilon$ = 0.3. **b** Examples of dislocation networks, triple junctions, and pileups occurring throughout the simulated volume. **c** Slip systems highlighted for visualization.

All 3D DDD simulations were performed using the MODELib package^54^ using strain-controlled compression on a single crystal of Nb oriented in the $\langle 123\rangle$ direction. The crystals were square cross section prisms with an edge length of $3000$ Burgers units and a height of $9000$ Burgers units. Temperatures in the 173–323 K range were used. All simulation results displayed in the main manuscript file were obtained at a strain rate of $\dot{\varepsilon }=718\,{{{\mbox{s}}}}^{-1}$, and additional data in the SOM was obtained at 7180 s^−1^. During post-analysis an avalanche event was specified as beginning when the measured continuous plastic strain rate was greater than the applied strain rate and ending when the continuous plastic strain rate dropped to below the applied strain rate. Dislocation network characteristics and statistics were evaluated as direct output from the DD simulations. Figure 8a demonstrates the full-size simulated DDD volume. Figure 8b provides several examples of observed dislocation impediments including dislocation pileups, triple junctions, and networking. Figure 8c shows the ascribed slip planes for the dislocations shown in Fig. 8a.

The dislocation mobility (i.e. the relationship between elastic forces and dislocation velocities) used in this work was developed specifically for bcc metals^42^, and includes a mixed thermally activated/viscous mobility for screw dislocations (both below and above the Peierls stress) and a viscous law for edge dislocations.

In terms of the makeup of the dislocation network, all the simulations were initially run with a 50/50 edge/screw distribution. After a dominance of screw segments in the plastic response was observed, we decided to bias the initial microstructure more towards the edge component to ensure the absence of a ‘preconditioning’ of the system towards a screw-dominated response with the 50/50 microstructure. For this, several simulations with a global dislocation character skewed towards the edge component in the initial configurations (60% edge/40% screw and higher) were conducted. We observed the same effective response (characterized by a screw dominance) regardless of the makeup of the initial microstructure, adding confidence to the general observations extracted from the simulations.

## Supplementary information

Supplementary Information

## Data Availability

The data that support the findings of this study are available from the corresponding author upon reasonable request.

## Code availability

The code used in this study is available from the corresponding author upon reasonable request.
