# Supplementary material for: Mild-to-wild plastic transition is governed by athermal screw dislocation slip in bcc Nb
Source: Nat Commun. 2022 Feb 23;13:1010. doi: 10.1038/s41467-022-28477-4 (PMC8866410; doi:10.1038/s41467-022-28477-4)
Supplement: Supplementary file 1 — Supplementary Information [file 41467_2022_28477_MOESM1_ESM.pdf]

Mild-to-wild plastic transition is governed by athermal screw dislocation slip in bcc Nb

Q. Rizzardi[1], C. McElfresh[2], G. Sparks[1], D.D. Stauffer[3], J. Marian[2], R. Maaß[1,4*]

[1]*Department of Materials Science and Engineering, University of Illinois at Urbana-Champaign, Urbana, IL 61801, USA*
[2]*Department of Materials Science and Engineering, University of California, Los Angeles, Los Angeles, California 90095, USA*
[3]*Bruker Nano Surfaces & Metrology, Hysitron Products, Eden Prairie MN 55344*
[4]*Federal Institute of Materials Research and Testing (BAM), Unter den Eichen 87, 12205 Berlin, Germany*

* robert.maass@bam.de

SUPPLEMENTARY ONLINE MATERIAL

This supplement contains additional details about the experiment and the simulation work behind this research. Supplementary Note 1 presents our noise determination process, and how we can reach an event-size resolution of 0.165 nm. Supplementary Note 2 describes in detail our calculation of strain-rate sensitivity temperature dependence and our comparison with literature data. Supplementary Note 3 presents the statistical analysis of simulation data and discusses it relative to the experimental data. Supplementary Note 4 explains in more detail the model developed by Kubin & Jouffrey [1] and how it allows us to determine an activation energy for the system as a function of temperature.

Supplementary Note 1: Noise determination routine

Considering that many of the recorded intermittent events are of nanometer scale and lower, it is paramount to establish a noise threshold value above which all detected events can be shown to be actual events rather than random noise fluctuations. Our approach starts with the recording of nanoindenter noise under an elastic load level of 200 μN for durations between 5 and 10 min at a DAR of 800 Hz to ensure a robust estimation of the noise distribution. The unfiltered displacement data (in nm) is then extracted and the absolute displacement between consecutive points is tallied. We find similar results for the TI-980 used at lower temperatures and the TI-950 used at room temperature: the noise follows a Gaussian distribution, with a mean $\mu = 2 \times 10^{-5}$ nm (taken to

be close enough to be approximated as zero) and standard deviation $\sigma = 0.21 \pm 0.01$ nm, depending on dataset and indenter setup. We take the higher value of $\sigma = 0.22$ nm that expresses our standard noise distribution.

This alone does, however, not result in a noise threshold: since intermittent events correspond to a sustained increase in measured displacement, we consider the total shift of the measured depth and compare it to the established noise envelope. We do this by considering the data before and after the timeframe where a potential event has been detected. On both ends we select at least 50 datapoints ($\approx$ 60 ms at 800 Hz), and for each dataset we apply the same noise calculation as above and generate a noise envelope of $\mu \pm 2\sigma$. Should the noise calculation of a dataset result in a tighter Gaussian distribution than the standard distribution defined above, we create the noise envelope using the parameters of the standard distribution instead. With the noise envelopes on each side of the detected event, we consider that a displacement resulting in a shift of at least one half of the width of the noise envelope is a sustained change in the system, and the event is retained as valid. Should the noise envelopes overlap more than this, the event is considered uncertain and rejected from the dataset. After this selection process, the retained events are further refined by Wiener filtering, which allows us to extract a smooth velocity profile and a corresponding peak velocity as described in the Methods Section. Filtering removes any undesired velocity subpeak that results from the standard noise distribution, and as such allows us to improve our event size even further. This entire routine returns a noise threshold valid for all experiments that we find to be 0.165 nm. Above this detection threshold, the point-to-point resolution of 0.006 nm sets the lower bound of a detectable size increment relative to 0.165 nm but depends also on a combination of the denoising procedure and the peak-velocity profile fitting, which is hard to quantify. Since the detection limit is small and the resolution is high (ca. 3.6% of the detection limit), possible surface irregularities or sample-surface contaminations may need to be considered. These would be an issue, if we were to analyze load-displacement discontinuities upon the initial contact (very low forces) between the sample and the tip. However, here we are resolving the abrupt length changes of a crystal at a fully established mechanical contact, of which the dynamical response is fully outlined in Ref. [2]. Under the mechanical contact during the plastic flow of the crystals, surface contaminations or possible surface irregularities are therefore not expected to play any role.

Supplementary Note 2: Strain-rate sensitivity calculation

The strain rate sensitivity (SRS) is a material-dependent property that we use here as a way to characterize our niobium crystal and compare experimental and simulation behavior: namely, we are interested in the temperature dependence trend of the SRS, indicated by $\frac{\partial m}{\partial T}$ with m being the SRS exponent. Previous literature (Refs. [3,4]) featured in Supplementary Figure 1 gives us an indication of the expected trends of in bulk crystals: a straightforward linear fit of $m = aT + b$ returns values of a ranging from 1.7 to 1.9.

In comparison, the temperature trends of m found for DD and our experimental data give values of $a = 2.9$ and 3.1, respectively. While the values are somewhat larger than the ones found in literature, the absolute values of m and the general upward trend are consistent with previous works. Moreover, the strong agreement between the DD trend and the experimental one indicates that both systems show the same relative sensitivity to strain rate as a function of temperature: as such, we believe their strain behavior at different temperatures can be compared despite the notable difference in strain values achieved in DD vs experiment. This is because the SRS exponent m gives the scaling of how plastic deformation relates to applied stress. Given the similar temperature dependence found in our experiments and simulations, our assumption about the correspondence between DD and experiments is justified. Regarding the constant part of the m vs T curve, which is admittedly different in both cases, this is less significant in terms of the experimental-vs-simulation comparison because dislocation densities, pillar geometries, and precise loading conditions are all (slightly) different in the simulations and the experiments.

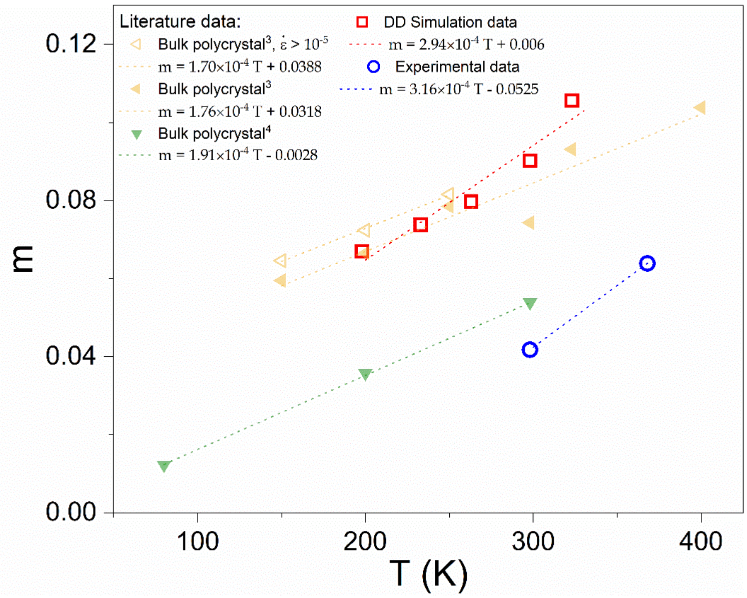

Supplementary Fig. 1. Temperature-dependent strain-rate sensitivity across experiments and simulations. Previous studies of the temperature dependence of strain-rate sensitivity in Nb crystals are found in Refs. [3,4] and added here for comparison purposes, showing that both the absolute values and the temperature dependence trends found for our experimental and simulation work fall within the expected ranges of m.

Supplementary note 3: Statistical distribution of DD data

The CCDF of DD data can be expressed in the same way it was done for the experimental data. Unlike what is seen in experiments, the DD data shows little temperature dependence in the shape of the CCDF: it continues to follow a truncated power-law distribution regardless of the temperature (Supplementary Figure 2a). However, we see a marked difference in the distribution at a higher strain rate of $7180\ s^{-1}$, with event size reaching up to 1 nm (Supplementary Figure 2b). This is not unexpected behavior: similar increase in strain rates have been shown experimentally to “drive” intermittent plasticity [5].

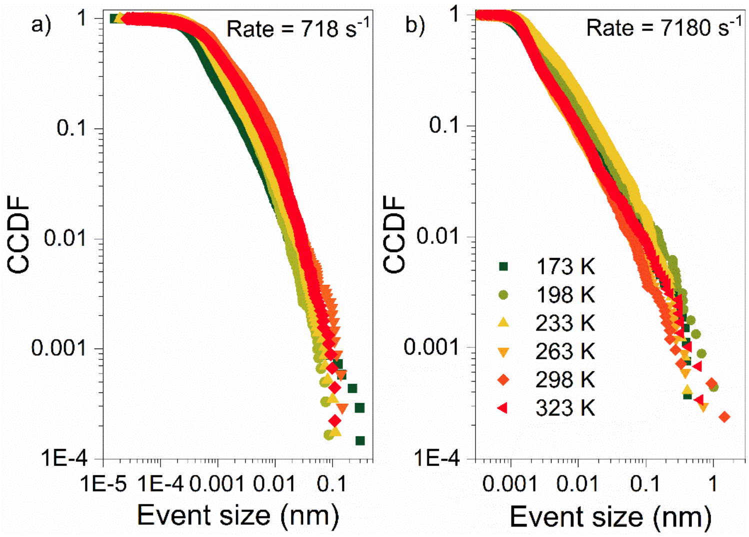

Supplementary Fig. 2. Temperature dependence of event sizes in DD simulation. Simulation work shows little temperature dependence of intermittent plasticity, whether we are considering a strain rate of **a.** $718\ \mathrm{s}^{-1}$ or **b.** $7180\ \mathrm{s}^{-1}$.

This intermittent plastic mode is the result of collective dislocation behavior, where a number of dislocation sources trigger a correlated motion in a local dislocation network, thus creating an avalanche of motion which is detected as "intermittent" due to the mismatch between the applied and the recorded strain rate. From there, we see that an avalanche with an absolute displacement may be detected as intermittent at relatively low strain rates but appears as smooth if the strain rate matches the avalanche in velocity and relative displacement. In other words, the size increase of events between both strain rates can be explained by the discrepancy between the absolute measure of dislocation displacement during intermittency and the relative measure of strain (and strain rate).

Supplementary note 4: Model of activation energy

The activation energy determined in Fig. 1b in the main text is the result of the work in Ref. [1], which offers an experimental model tying activation energy ΔG, stress τ and activation volume v^* as a function of temperature T and can be summarized by the following set of equations:

$$v = v^{*}\left(1 - \frac{\tau^{*}}{\tau_i}\right) \tag{1}$$

$$\Delta G = CkT = \frac{v^{*}\tau_i}{2}\left(1 - \frac{\tau^{*}}{\tau_i}\right)^2 \tag{2}$$

$$\tau_i - \tau = -\tau^{*} = AT^{1/2} \tag{3}$$

With C and A constants. Once τ_i and A have been determined by fitting the values of stress found experimentally (as per Fig. 1b), the values can be used to determine ΔG as a function of τ^{*} or τ (as per the inset of Fig. 1b) or as a function of T. At $T = 298$ K we find $\Delta G \approx 0.28$ eV.

Supplementary References

1 Kubin, L. P. & Jouffrey, B. Etude de la déformation plastique de monocristaux de niobium de haute pureté a basse température. *Philos. Mag.* **27**, 1369-1385, doi:10.1080/14786437308226893 (1973).

2 Sparks, G., Phani, P. S., Hangen, U. & Maaß, R. Spatiotemporal slip dynamics during deformation of gold micro-crystals. *Acta Mater.* **122**, 109-119, doi:10.1016/j.actamat.2016.09.026 (2017).

3 Briggs, T. L. & Campbell, J. D. The effect of strain rate and temperature on the yield and flow of polycrystalline niobium and molybdenum. *Acta Metall.* **20**, 711-724 (1972).

4 Nemat-Nasser, S. & Guo, W. Flow stress of commercially pure niobium over a broad range of temperatures and strain rates. *Mater. Sci. Eng., A* **284**, 202-210 (2000).

5 Sparks, G. *et al.* Avalanche statistics and the intermittent-to-smooth transition in microplasticity. *Physical Review Materials* **3**, doi:10.1103/PhysRevMaterials.3.080601 (2019).
